# Cutaneous Neuroendocrine Carcinoma of the External Auditory Canal: A Case Report and Review of the Literature

**DOI:** 10.1155/2012/941065

**Published:** 2012-08-05

**Authors:** Yi-Ke Li, Fang-Lu Chi, Shu-Yi Wang, Wu-Qing Wang, Juan-Mei Yang, Yi-Bo Huang

**Affiliations:** ^1^Department of Otolaryngology, School of Medicine, Vanderbilt University, Nashville, TN 37232, USA; ^2^Department of Otology and Skull Base Surgery, Eye Ear Nose and Throat Hospital, Fudan University, Shanghai 200031, China; ^3^Department of Pathology, Eye Ear Nose and Throat Hospital, Fudan University, Shanghai 200031, China

## Abstract

Cutaneous neuroendocrine carcinoma (cNEC) is rarely seen in the external ear. In this paper, we newly describe a patient with cNEC in his right external auditory canal, followed by a further discussion on the clinical features, diagnosis, and treatments of cNEC of the external ear. A review of the literature showed that cNEC of the external auditory canal generally presents as asymptomatic and that pathology yields the most confirmative diagnosis. A wide resection with adjuvant radiotherapy and chemotherapy is recommended. The overall prognosis of this condition is poor.

## 1. Introduction

Neuroendocrine carcinoma (NEC) constitutes a heterogeneous group of neoplasms that have been postulated to originate from a common precursor cell population. Though commonly associated with the gastrointestinal tract and bronchopulmonary system, a substantial number of these tumors originate in less common anatomical sites and can range from indolent, unrecognized entities to highly active, metastatic secretory tumors. In 1972, Toker [1] first described an uncommon skin lesion which presented as a trabecular pattern of tumor cell growth, later named cutaneous neuroendocrine carcinoma (cNEC). Most of these cases are known as Merkel cell carcinoma (MCC), which is generally considered to originate from Merkel cells in the basal layer of the epidermis. Much less often, cNEC is diagnosed in the external ear. To our best knowledge, only 23 cases of cNEC of the external ear have been reported [2–5], and only 3 of them were found in the external auditory canal [6, 7]. 

In this paper, we newly describe a patient with cNEC in his right external auditory canal, followed by a further discussion on the clinical features, diagnosis, and treatment of cNEC of the external ear.

## 2. Case Report

**Figure 1:**
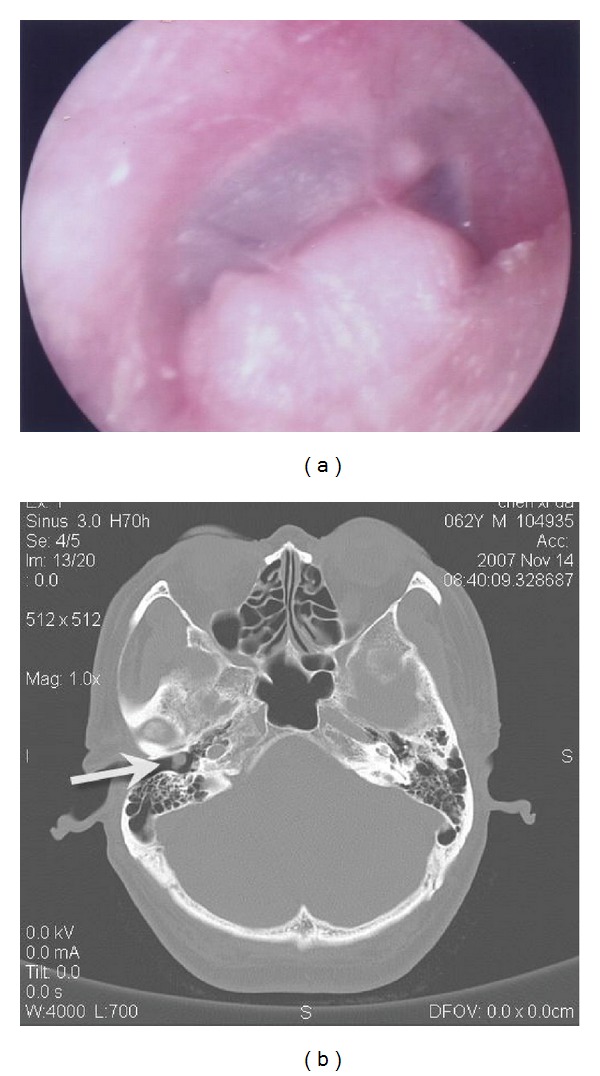
Auxiliary examinations including (a) myringoscope which showed a flesh-colored mass in the anterior and inferior walls of deep right external auditory canal, near the tympanic membrane. (b) Axial cranial CT which presented a well-circumscribed soft tissue mass with homogeneous density (white arrow) in the right external auditory canal.

A 62-year-old, otherwise healthy male was referred to our hospital in November 2007 with a two-year history of a painless mass in his right external auditory canal. The patient had surgical history positive for removal of a small keratoacanthoma in his right external auditory canal in 2001. Since then he had not experienced any discomfort or symptoms until a mass was found in his right external auditory canal during a routine physical exam in 2005. As the lesion was asymptomatic at that time, the patient refused immediate resection proposed by his doctor and decided to postpone treatment until after he retired. Myringoscope revealed a mass in the deep right auditory canal near the tympanic membrane (Figure 1(a)). The mass was approximately 1 centimeter in diameter, smooth in appearance, and with no sign of bleeding. CT scan revealed a well-circumscribed, slightly enhanced solid mass in the inferior wall of the right external auditory canal without bony erosion (Figure 1(b)). Facial electromyography, vestibular function tests, and pure tone audiogram all came back normal. 

The mass was excised through a microscope-guided surgery, during which we removed the flesh-colored mass that was adherent to the surface of tympanic membrane and preserved the canal wall and the tympanic membrane. The excised mass was then sent to pathology.

### 2.1. Pathologic Features

**Figure 2:**
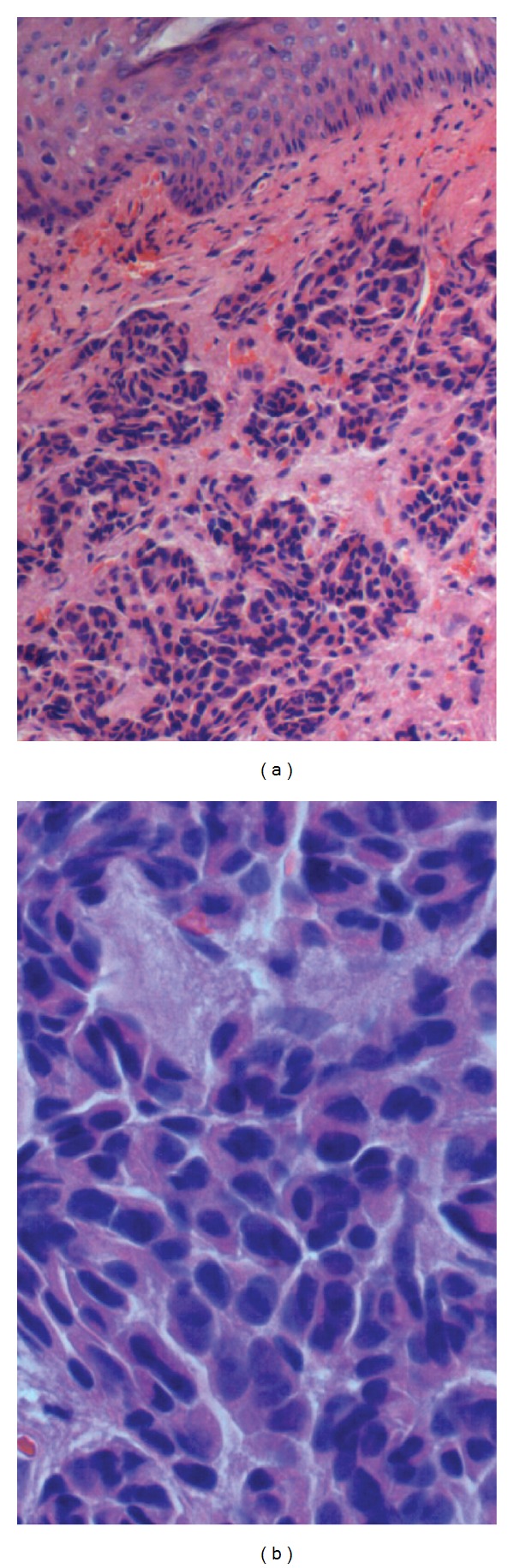
Pathological examination revealed (a) irregular cell trabeculae or clusters lying in lamina propria, without involving the epidermic layer (HE stain ×100); (b) tumor cells which had a moderately differentiated appearance, containing pleomorphic nuclei with increased mitotic activity (HE stain ×400).

Tissues were fixed in 10% formalin, imbedded by paraffin, routinely sliced, and HE stained. To our surprise, through light microscopy, we found that the tissues demonstrated cord, trabeculae, or cluster proliferations of moderately differentiated cells in dermal and proper layers, with pleomorphic nuclei and inconspicuous nucleoli, in addition to frequent mitoses. There were also scattered cell fragments with pyknotic nuclei, indicating apoptosis. The cuticular layer was not involved (Figures 2(a) and 2(b)).

**Figure 3:**
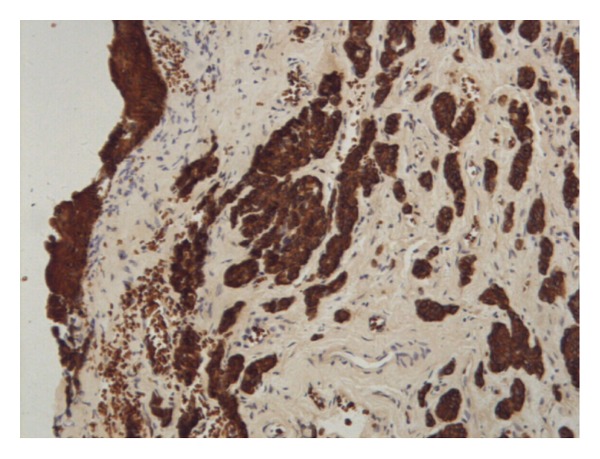
The tumor cells were strongly positive for cytokeratin (CK stain ×100).

**Figure 4:**
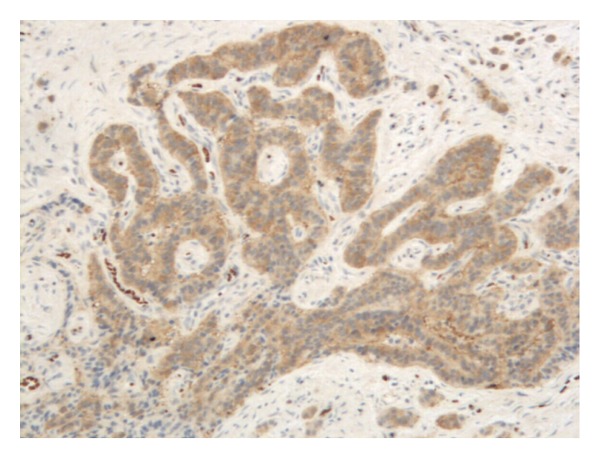
The tumor cells were strongly positive for NSE (NSE stain ×100).

In order to confirm the diagnosis, immunohistochemistry was carried out on paraffin sections using standard techniques with antibodies against cytokeratin (CK), epithelial membrane antigen (EMA), vimentin, neurone specific enolase (NSE), S-100 protein, smooth muscle actin (SMA), synaptophysin, glial fibrillary acidic protein (GFAP), and chromogranin (ChG). The result revealed strongly positive for CK and NSE (Figures 3 and 4) and moderate positive for vimentin, showing a pattern consistent with a neuroendocrine tumor.

As we did not see normal tissues on certain regions of the tumor margin, a secondary surgery was proposed for complete clearance of the tumor. 

### 2.2. Further Treatment and Progress

The patient underwent a PET scan two days after initial surgery, which did not show evidence of local lymph node involvement or metastasis. Nine days after the first surgery, the patient underwent a sleeve mastoidectomy and tympanotomy, during which we removed the tympanic membrane and performed a sleeve excision of skin and bone in the right external auditory canal. The tympanic cavity was clean. A temporoparietal fascial flap was used to reconstruct tympanic membrane and a postauricular free flap was created to cover the external auditory canal. One week after the second surgery, the patient received a chemotherapy combined of cisplatin, calcium folinate, and tegafur for a three-day course. In addition, a radiotherapy protocol was added, that a level of 200 cGy radiation was delivered each time for a total of 30 times. The patient did not experience any signs of adverse reaction throughout the entire postoperative treatment. He completed the treatment three months after his admission, and was asked for routine followup every two months in the first six months and every half a year thereafter. The total follow-up period was 2 years, no evidence of tumor recurrence was found in the patient.

## 3. Discussion

cNEC that occurs in the head and neck is much more commonly seen in cheeks, nose, and larynx than in the external ear [8]. Up to now, the majority of case reports concerning cNEC of the external ear are diagnosed as MCC, which possesses similar clinical, pathological, and immunohistochemical characteristics to small cell neuroendocrine carcinoma. However, cases of both cutaneous endocrine adenoma [3] and large cell cNEC [5] occurring in external ear have also been reported, which indicates that MCC is not the only type of neuroendocrine tumor of the external ear. Also, considerable debates on the proper name of this tumor still exist because no concrete evidence has been found to prove whether cNEC is derived from epidermal Merkel cells, dermal neuroendocrine cells, or pluripotent dermal stem cells. Therefore, we use cNEC in this paper instead of MCC, although they are believed to be synonymous on various occasions.

It is generally believed that the risk factors for cNEC include exposure to radiation, immunosuppression, and old age. Yet only the age factor was presented in our patient. Consistent with most case reports that we identified, our patient presented no obvious symptoms or signs. Unlike NEC occurred in the lung or pancreas, where secretion of tumor cells might lead to noticeable symptoms, cNEC rarely causes carcinoid syndrome. This is perhaps due to the fact that the tumor cells of cNEC are usually functionally silent.

In this case, evidences including patient's medical history and preoperative tests indicated that the mass was most likely benign, but the more conclusive postoperative pathology confirmed it as malignant. Clinical signs and symptoms were negligible so that this tumor may lack features distinct from other tumors that commonly occur in the external ear, such as basal cell carcinoma, melanoma, lymphoma, and ceruminous gland carcinoma. In fact, early diagnosis of cNEC of the external ear should be a challenge for both patients and clinicians because it often appears as a tiny, indolent, and unspecific entity. However, once there is local lymph node involvement or metastasis, which are not rarely seen until the later stage of this tumor, the overall prognosis is far worse due to the advanced presentation of the disease [9]. Therefore, an early biopsy or resection should be recommended whenever a growing, solid mass is found in the external ear. Meanwhile, a PET scan is helpful if metastasis is suspected [9].

Pathologic examination and immunohistochemistry are essential to confirm the diagnosis of cNEC and the latter one may provide useful information for the differential diagnosis of other tumors that appear similar under microscope. Our case refers to a moderately differentiated histotype, as characterized by tumor cells with a relatively small to medium-sized uniform appearance, in addition to pleomorphic nuclei and inconspicuous nucleoli. There were mitotic figures and scattered cell fragments with pyknotic nuclei, indicating apoptosis. It would also be helpful to confirm the diagnosis of cNEC if neurosecretory granules present within the cytoplasm of tumor cells.

Surgical intervention is generally accepted as the standard treatment of care and the only approach that can achieve a cure in patients with NEC at any site. The most common method is wide resection of the primary tumor. The role of prophylactic neck dissection in the standard treatment of cNEC of the external ear remains unclear, but may reduce the recurrence rate. In addition, adjuvant treatments of radiotherapy to the primary site and primary nodal chains, as well as combination chemotherapy, are also recommended because they have shown considerable short-term and long-term control rates in clinical trials [9, 10]. Our patient underwent a wide excision by sleeve mastoidectomy and tympanotomy but we did not perform a neck dissection, because there was no evidence of local lymph node involvement. Adjuvant chemotherapy and radiotherapy were also administered in order to diminish the risk of recurrence. Although the efficacy of radiopharmaceutical therapy with radionuclide-targeting agents, such as ^131^I-MIBG, ^111^In-octreotide, or ^90^Y-lanreotide, was observed in NECs of digestive system [10], currently there is no published literature on its therapeutic effectiveness for cNEC of external ear.

Patient's age and physical condition, tumor size, tumor staging, histological differentiation, and treatment are all prognosis factors for cNEC. Particularly, TNM staging is considered to be the most consistent and independent predictor for survival rate in MCC cases [9]. According to the staging standard [11], the tumor was in Stage I in our case. Generally, the overall prognosis for cNEC of the external ear is poor [2], metastasis and recurrence were usually seen. At the time when this paper was written, there was no sign of recurrence in our patient, and we believe this is attributable to an early staging of tumor and a thorough treatment protocol. Spontaneous regression of cNEC of the external ear has been reported [12].

## 4. Conclusion

cNEC rarely occurs in the external ear and usually presents as MCC. Clinical features alone do not lead to an easy diagnosis. Therefore, biopsy or excision is essential for early detection of cNEC if an indolent mass is found in the external ear. Only histological diagnosis is conclusive and the contributions of pathologic examination and immunohistochemistry are crucial. A wide resection with adjuvant radiotherapy and chemotherapy may minimize the risk of tumor recurrence. The overall prognosis of cNEC of the external ear is poor, but several prognosis factors can influence its final outcome.
